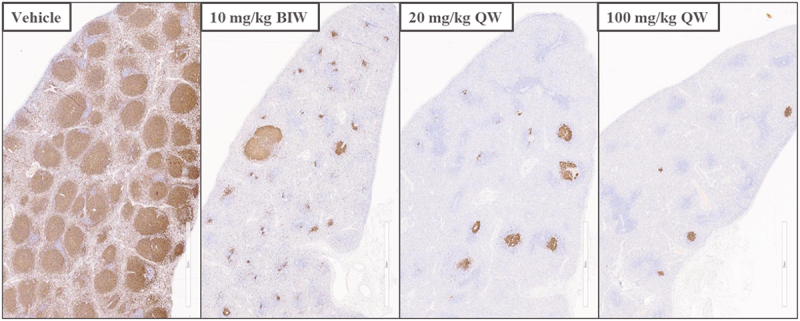# Correction

**DOI:** 10.1080/19420862.2024.2354626

**Published:** 2024-05-12

**Article title: CC-96673 (BMS-986358), an affinity-tuned anti-CD47 and CD20 bispecific antibody with fully functional fc, selectively targets and depletes non-Hodgkin’s lymphoma**

**Authors**: Dan Zhu, Haralambos Hadjivassiliou, Catherine Jennings, David Mikolon, Massimo Ammirante, Sharmistha Acharya, Jon Lloyd, Mahan Abbasian, Rama Krishna Narla, Joseph R. Piccotti, Katie Stamp, Ho Cho, and Kandasamy Hariharan

**Journal**: *mAbs*

**DOI**: 10.1080/19420862.2024.2310248

The author discovered an error in one of the image [copied and pasted the same image for both 20 mg/kg and 100 mg/kg groups by accident]. The amended version of this figure is now shown below. The authors sincerely apologize for this error.

**Corrected version of Figure 6C:**

**Figure:**